# Selective covalent capture of collagen triple helices with a minimal protecting group strategy†

**DOI:** 10.1039/d1sc06361h

**Published:** 2022-02-17

**Authors:** Le Tracy Yu, Jeffrey D. Hartgerink

**Affiliations:** Rice University, Department of Chemistry and Department of Bioengineering Houston TX 77005 USA jdh@rice.edu

## Abstract

Collagens and their most characteristic structural unit, the triple helix, play many critical roles in living systems which drive interest in preparing mimics of them. However, application of collagen mimetic helices is limited by poor thermal stability, slow rates of folding and poor equilibrium between monomer and trimer. Covalent capture of the self-assembled triple helix can solve these problems while preserving the native three-dimensional structure critical for biological function. Covalent capture takes advantage of strategically placed lysine and glutamate (or aspartate) residues which form stabilizing charge–pair interactions in the supramolecular helix and can subsequently be converted to isopeptide amide bonds under folded, aqueous conditions. While covalent capture is powerful, charge paired residues are frequently found in natural sequences which must be preserved to maintain biological function. Here we describe a minimal protecting group strategy to allow selective covalent capture of specific charge paired residues which leaves other charged residues unaltered. We investigate a series of side chain protecting groups for lysine and glutamate in model peptides for their ability to be deprotected easily and in high yield while maintaining (1) the solubility of the peptides in water, (2) the self-assembly and stability of the triple helix, and (3) the ability to covalently capture unprotected charge pairs. Optimized conditions are then illustrated in peptides derived from Pulmonary Surfactant protein A (SP-A). These covalently captured SP-A triple helices are found to have dramatically improved rates of folding and thermal stability while maintaining unmodified lysine–glutamate pairs in addition to other unmodified chemical functionality. The approach we illustrate allows for the covalent capture of collagen-like triple helices with virtually any sequence, composition or register. This dramatically broadens the utility of the covalent capture approach to the stabilization of biomimetic triple helices and thus also improves the utility of biomimetic collagens generally.

## Abstract 2

A minimal protecting group strategy is developed to allow selective covalent capture of collagen-like triple helices. This allows stabilization of this critical fold while preserving charge–pair interactions critical for biological applications.

## Graphical abstract

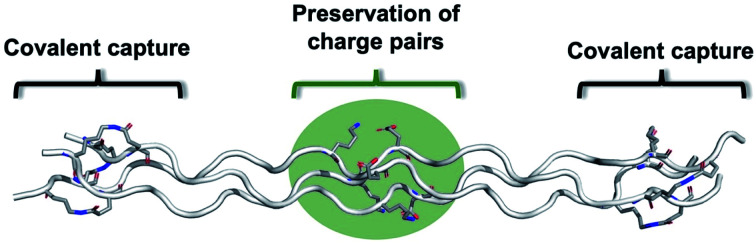

## Introduction

Covalent bonds are frequently used by proteins for stabilization of the architecture pre-assembled from non-covalent interactions. One of the best known examples is human insulin, a dimer linked by two interchain disulfide bonds.^1–4^ Similarly, ribonuclease is stabilized by the formation of four disulfide bonds.^5,6^ More recently it was found that the NC1 domain of type VI collagen is stabilized by a sulfilimine bond formed between the side chains of methionine and lysine.^7–9^ These systems, and many others, take advantage of noncovalent interactions for control of the folding of their three dimensional structure and subsequent covalent bond formation for stabilization, a process known as covalent capture.^10,11^

Inspired by Nature, researchers have utilized covalent capture strategies to stabilize designed self-assembling systems. Alpha helical,^12^ beta sheet^13–17^ and polyproline type 2 secondary^18–21^ structures have been prepared by this self-assembly followed by covalent capture approach.

Our group recently developed a covalent capture technique for collagen triple helices in which isopeptide bonds are formed between the side chains of lysine and aspartate or glutamate.^20–22^ These engineered collagens were found to have improved thermal stability and an increased rate of folding while perfectly preserving the native triple helical fold of the assembled peptides.

**Fig. 1:**
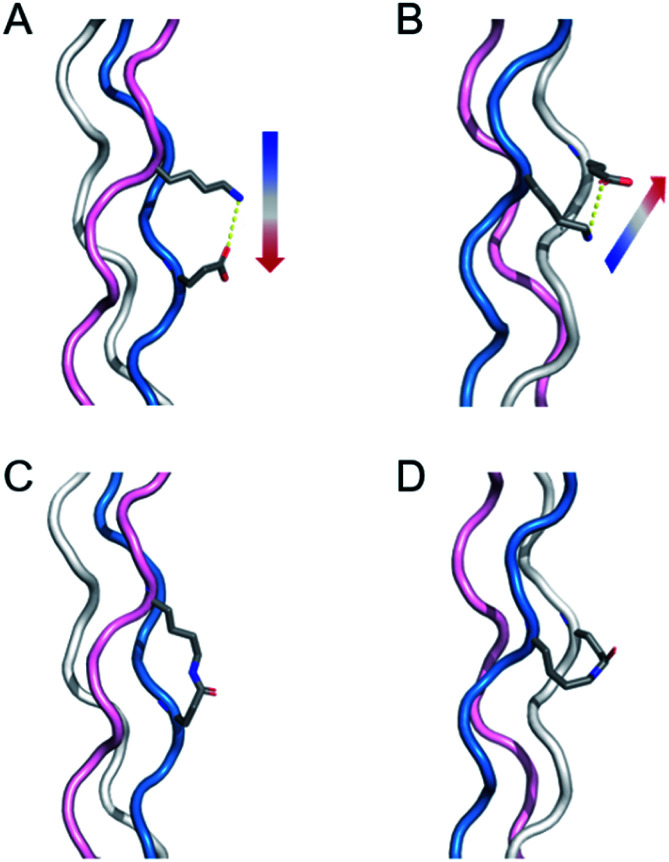
Illustration of salt-bridges in collagen triple helices between the side chains of Lys and Glu. (A) Axial interaction. The figure is generated from PDB 3t4f.^27^ (B) Isopeptide bond formation by covalently capturing the axial salt-bridge. Figure is generated from PDB 6vzx.^21^ (C) Lateral interaction. (D) Isopeptide bond formation by covalently capturing the lateral salt-bridge. Models and figures were generated with PyMOL 2.5.1.

Collagen is the most abundant protein in mammals and has a characteristic triple helical structure formed from three peptides adopting a polyproline type 2 structure.^23–26^ Covalent capture is particularly important for collagen-like triple helices as they are well known to have very slow rates of folding (hours to weeks) and only moderate thermal stability (frequently below physiological temperature), two problems that covalent crosslinking can overcome. Our previous work first directed triple helix self-assembly with selective charge paired hydrogen bonding interactions and then covalently linked these amino acids to form isopeptide bonds. This approach only works when the lysine and glutamate residues are in one of two specific sequential geometries with respect to one another forming axial (Fig. 1a) or lateral (Fig. 1b) interactions. Their closeness in space results in a proximity directed selectivity during amide bond formation which results in all those axial and lateral pairs reacting to the exclusion of any other potential partners (Fig. 1c and d). This approach works well as long as no other lysine–glutamate or lysine–aspartate axial or lateral pairs exist that need to be maintained in their supramolecular, zwitterionic state. However, due to the frequency of this interaction in biologically interesting sequences, this can be a significant limitation of the approach. Therefore, it is important to develop a method that can selectively form a covalent bond using charge–pair interactions in collagen triple helices, yet avoid modification of critical, naturally charged residues.

A straightforward solution to this problem is to protect the side chains of specific charged amino acid residues in the natural sequences while leaving the amino acids which are desirable to crosslink unprotected. After triple helix folding and the iso-peptide amide bond formation, the protecting groups can be removed. However, a successful approach requires that (1) protecting groups do not adversely impair helix solubility nor (2) disrupt the structure and stability of the triple helix. (3) They must also prevent unwanted isopeptide amide bond formation while (4) not interfering with distal reactivity. Finally, (5) they must be easily removable under relatively mild conditions in good yield. Here we develop methods for selective covalent capture in the presence of multiple charge–pair interactions through the use of a minimal sidechain protection strategy.

## Results and discussion

### Peptide design

**Scheme 1:**
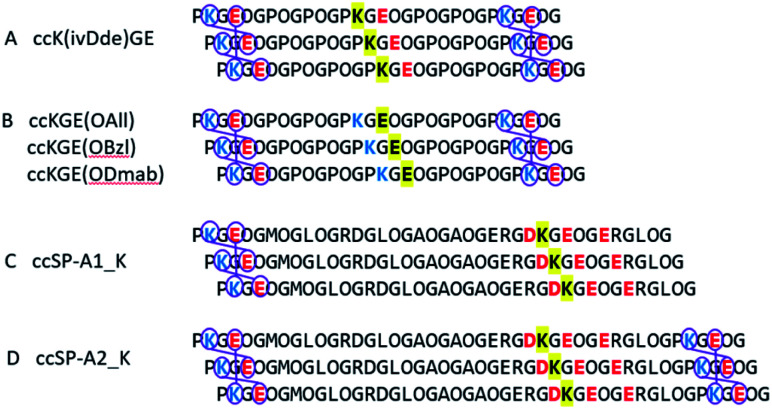
Selective covalent capture of collagen triple helix with protecting groups. The designed triple helices are expected to have a canonical registration. “O” represents (4*R*)-hydroxyproline. Blue and red colors represent positively and negatively charged amino acid residues, respectively. Yellow highlights the residues that will be protected by Fmoc-orthogonal protecting groups, specifically ivDde on the side chain of lysine, OAll, OBzl, and ODmab on the side chain of glutamic acid. (A and B) are template triple helices with different protecting schemes (Table S1†). (C and D) are modified peptide sequences from protein rat SP-A. Triple helix (C and D) have 6 and 3 iso-peptide bond formations, respectively. The sequences were obtained from universal protein knowledgebase.^42^

Two protecting schemes are available: protection of positively or negatively charged amino acid residues. In principle, all charged amino acids could be protected, but this is synthetically more cumbersome and likely would lead to poor aqueous solubility and failure of the helix to fold. Therefore, our goal was to use the minimum number of protecting groups possible. Based on this, either the target lysine(s) or target glutamate(s) were protected, but never both. Scheme 1 shows the peptides prepared. Model peptide A was prepared with a single lysine protected while model peptide B was prepared with a single glutamate protected. Lysine was protected by ivDde which can be removed with 2% w/v hydrazine or 1 M hydroxylamine.^28,29^ Glutamate was protected by OAllyl, OBzl or ODmab. Effective chemical removal of allyl ester from peptides frequently involves the use of palladium catalysts.^30–32^ Although the development of advanced catalysts can reduce the catalyst's toxicity, the costs remain high and catalyst recovery is problematic. Benzyl esters can be removed by catalytic hydrogenation or saponification.^33,34^ ODmab protecting group is readily removable with 2% w/v hydrazine, but cyclization side reactions frequently occur.^29,35,36^ In this work, we have optimized deprotection reaction conditions with the template triple helices for low cost, high yield under aqueous conditions. These methods were then applied to the selective covalent capture of significantly more complex and chemically diverse peptides from SP-A in which (PKGEOG) motifs were added to the termini of the SP-A sequence to facilitate covalent capture (Scheme 1c and d). SP-A (pulmonary surfactant protein A) is a member of a family of proteins known as defense collagens^37^ which include collectins,^38^ C1q,^39^ ficolins,^40^ and others, which play critical roles in host-defense functions in mammals while contain collagen-like domains with complex amino acid sequences with predicted low thermal stability.^41^ Specific charged amino acid residues that are involved in the charge–pair interactions in the natural sequences were protected (highlighted in yellow). The SP-A derived triple helix has four internal salt-bridges in total and a lysine residue is potentially involved in all the salt bridges. Therefore, protecting the side chain of this specific lysine residue is the most economic strategy. Alternatively, if in a triple helix, there are more positively charged than negatively charged amino acid residues involved in salt bridges, protecting the negatively charged residues would be the most appropriate approach.

### Folding of collagen triple helices with protecting groups

**Fig. 2:**
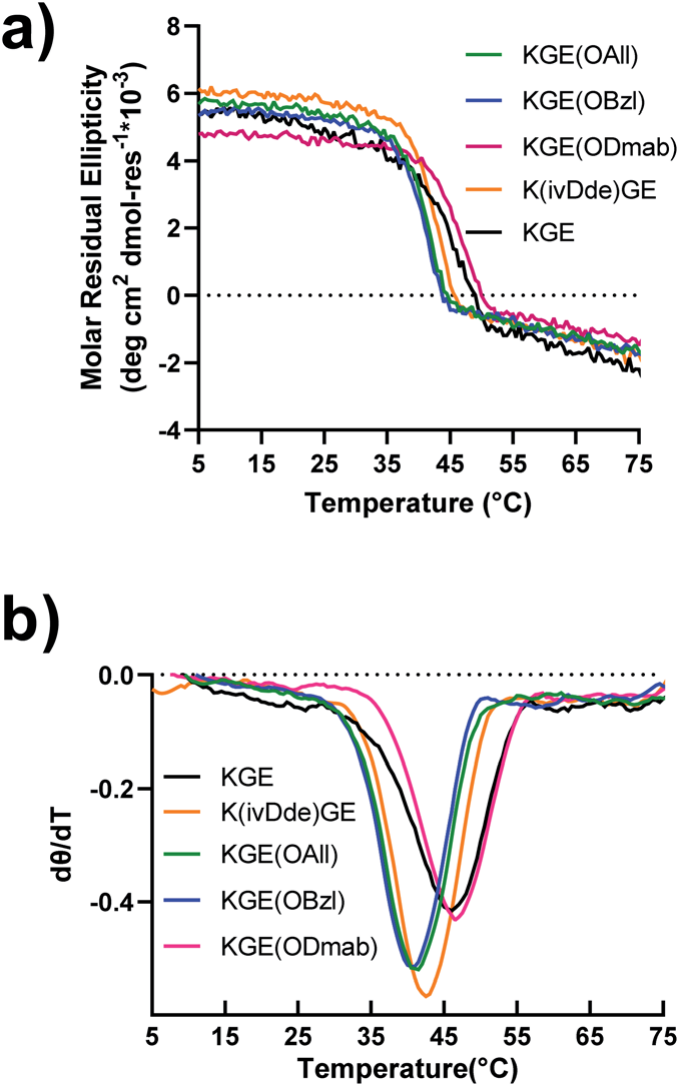
Circular dichroism of template collagen triple helices with protecting groups. (a) The melting (denaturation) curves measured at the wavelength of 225 nm. (b) The first-order derivative curves of the corresponding melting curves.

Peptides were prepared by solid phase synthesis using an FMOC strategy as previously described by us.^22,43,44^ Detailed methods are available in the ESI.† The prerequisite for covalent capture is the successful folding of the supramolecular collagen triple helices. Therefore, this requires the protecting groups on collagen triple helices cause no, or only minor, destabilizing effects on the self-assembly. We tested the influence of side chain protection with ivDde, ODmab, OAllyl and OBzl groups on the thermal stability of the tripe helices. Surprisingly, all the template peptides (protected model peptides A & B) with their corresponding protecting groups on the internal lysine or glutamate folded into triple helices, showing characteristic CD spectra with a maximal ellipticity at approximately 225 nm (Fig. S16†). Thermal unfolding experiments of the triple helices monitored at 225 nm (Fig. 2a) were performed and the minimum of the first derivative of the melting data (Fig. 2b) was used to assign the melting temperature of the triple helix. The unprotected KGE and the Dmab-protected KGE triple helices were found to have the highest melting temperatures of 45.5 °C. The ivDde, OBzl and OAllyl-protected triple helices have slightly lower melting temperatures of 42.5, 41 and 41 °C, respectively. Thus, we concluded that none of the protecting groups caused large destabilizing effects on triple helices that would preclude their further investigation.

The model peptide triple helices bearing protecting groups were covalently captured using the carboxylate activating reagents EDC and HOBt. Detailed methods are available in the ESI.† Briefly, the self-assembled, triple helical peptide was mixed with EDC and HOBt reagents in a ratio of desired isopeptide bonds/EDC/HOBt of 1 : 40 : 4. Each covalent capture reaction was monitored for 4 days. The formation of isopeptide bonds were assessed with mass spectra (Fig. S10†). Mass spectra suggest that (1) the triple helices with the protecting groups have been successfully covalently captured forming six isopeptide bonds and (2) the protecting groups remain unaffected during the covalent capture process. The covalently captured triple helices were then tested for side chain deprotection.

### Removal of the protecting groups from model triple helices

Several deprotection conditions were tested on peptide monomers and covalently captured trimers (Table 1). In some cases the conditions that worked smoothly for monomer peptides resulted in incomplete deprotection for the trimer samples. For example, using the condition of 25 eq. [Pd(All)Cl]_2_, 25 eq. GSH in 100 mM MES buffer, 37 °C, 30 min,^31^ we were able to achieve quantitative deprotection of the OAllyl group from monomer peptides, but similar conditions, even with elongated reaction time or higher reaction temperatures, resulted in incomplete deprotection of covalently captured triple helices. The deprotection conditions for Dmab that worked well for monomer peptides caused significant glutarimide/pyroglutamyl side product formation.^35,36^ We suspect that in the triple helix conformation, the amide proton of the adjacent strand can be close to the glutamate with Dmab protecting group, resulting in a geometry favourable for the cyclization reaction while this scenario happens less frequently in monomer peptides. In contrast to the above difficulties, we found that the simple saponification of benzyl and allyl esters worked surprisingly well. 80 mM K_2_CO_3_ at pH 11 was found to deprotect both the OAllyl and OBzl groups with high yield giving a clean mass spectrum of the deprotected trimer.

**Table 1:** Deprotection condition optimization^a^

Starting material	Entry	Conditions	Yield (%)
K(ivDde)GE	1	0.5 M NH_2_OH, 1 h, r.t.	96.9
KGE(OAll)	2	(1) 25 eq. [Pd(All)Cl]_2_, 25 eq. GSH, 37 °C, 30 min	97.6
		(2) 40 eq. DTT, centrifuge	
KGE(OBzl)	3	Pd black, H_2_, 12 h	78.5^b^
	4	70 mM NaOH, 1 h, 45 °C, pH 10–11	91.5
	5	80 mM K_2_CO_3_, 1 h, 45 °C, pH 10–11	97.4
KGE(ODmab)	6	2% w/v hydrazine, 30 min	>99
	7	0.5 M NH_2_OH, 2 h	>99
ccK(ivDde)GE	8	0.5 M NH_2_OH, 16 h, r.t.	90.1
ccKGE(OAll)	9	(1) 25 eq. [Pd(All)Cl]_2_, 25 eq. GSH, 45 °C, 12 h	30.0^c^
		(2) 40 eq. DTT, centrifuge	
	10	80 mM K_2_CO_3_, 2 h, 45 °C, pH 10–11	80.2
ccKGE(OBzl)	11	70 mM NaOH, 2 h, 45 °C, pH 10–11	n.d.^d^
	12	80 mM K_2_CO_3_, 2 h, 45 °C, pH 10–11	82.2
ccKGE(ODmab)	13	2% w/v hydrazine, 2 h	Trace^e^
	14	0.5 M NH_2_OH, 6 h	Trace^e^

a See the ESI for full reaction conditions.

b Methylated side products observed.

c The rest 70% is mostly the side product of covalently captured KGE trimer with one extra allyl ester group resulted from incomplete deprotection.

d The reaction condition is too strong which resulted in triple helix bound to multiple salts. The maldi mass spectrum shows salted trimer peaks of low resolution.

e The majority is side product of glutarimide/pyroglutamyl formation according to maldi mass spectrum.

**Fig. 3:**
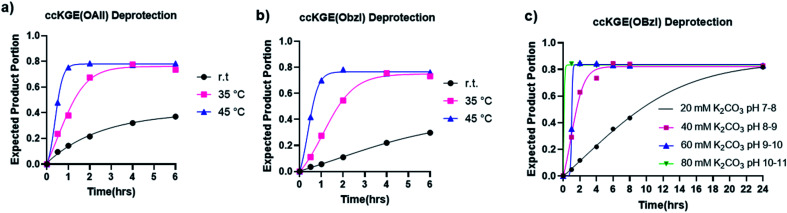
Hydrolysis kinetics of allyl and benzyl esters from covalently captured collagen triple helices ccKGE(OAll) and ccKGE(OBzl). (a) Allyl ester hydrolysis with 80 mM K_2_CO_3_ at different temperatures. (b) Benzyl ester hydrolysis with 80 mM K_2_CO_3_ at different temperatures. (c) Benzyl ester hydrolysis at 45 °C with K_2_CO_3_ solutions of different basicity.

The hydrolysis of esters is one of the easiest and most classic reactions but is rarely used in peptide synthesis. We have tested the hydrolysis kinetics of benzyl and allyl esters at different reaction conditions (Fig. 3). We first tested the allyl and benzyl esters hydrolysis with 80 mM K_2_CO_3_ at room temperature, 35 °C and 45 °C with the template triple helices. The reaction progress was followed with UPLC (Fig. S13 and S14†). We calculated the fraction of expected deprotected product by integrating the product peak area. According to our data, at 45 °C, the reactions achieve completion for OAllyl and OBzl removal after 1.5 and 2 h of reaction, respectively. At 35 °C, the reaction reaches completion after approximately 4.5 h for both OAllyl and OBzl removal. We observed less than 40% desired product at r.t. after 6 h reaction. After the reaction completion, we can obtain >80% of desired product and the rest was confirmed to be side product with one extra water loss according to the UPLC trace and mass spectra.

We then tested the deprotection at different pH conditions at 45 °C with K_2_CO_3_ using benzyl ester as an example (Fig. 3c and S15†). The results suggest that the hydrolysis can occur at very mild basic condition of pH 7–8 but it takes up to 24 h for the hydrolysis to reach completion. The deprotection works more efficiently at more basic conditions. According to our results, the hydrolysis of benzyl ester reaches completion after 6, 2, and 1.5 h of reactions with 40 mM, 60 mM, and 80 mM K_2_CO_3_, respectively.

The deprotection of ivDde protecting group from the side chain of lysine residue with 0.5 M NH_2_OH also worked successfully.

**Fig. 4:**
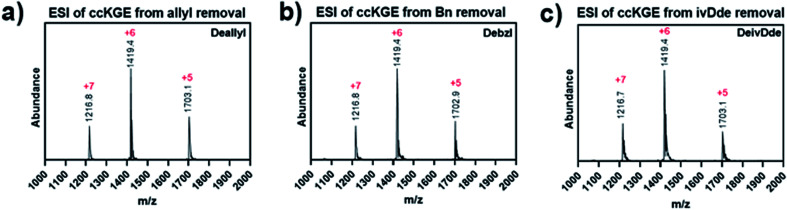
ESI Mass spectrum of covalently captured KGE template triple helices. The triple helices were removed of (a) allyl ester, (b) benzyl ester and (c) ivDde protecting groups.

After optimization, we achieved complete deprotection of ivDde, OAllyl and OBzl in covalently captured triple helices or monomeric peptides with conditions 8, 10 and 12, respectively (Table 1 and Scheme S1†). It should be noted that in all cases, before deprotection of the covalently captured triple helices, the solution was filtered with a 3K Dalton MWCO spin column to remove excess EDC and HOBt. Without this step additional water loss, presumably from unwanted isopeptide bond formation resulted. In the optimized conditions, the mass spectrum of the covalently captured trimer after deprotection show the correct mass peak with six water losses, indicating the successful covalent capture forming six isopeptide bonds and subsequent complete deprotection (Fig. 4).

### Increase of the triple helices thermal stability and refolding kinetics by covalent capture of the template triple helices

**Fig. 5:**
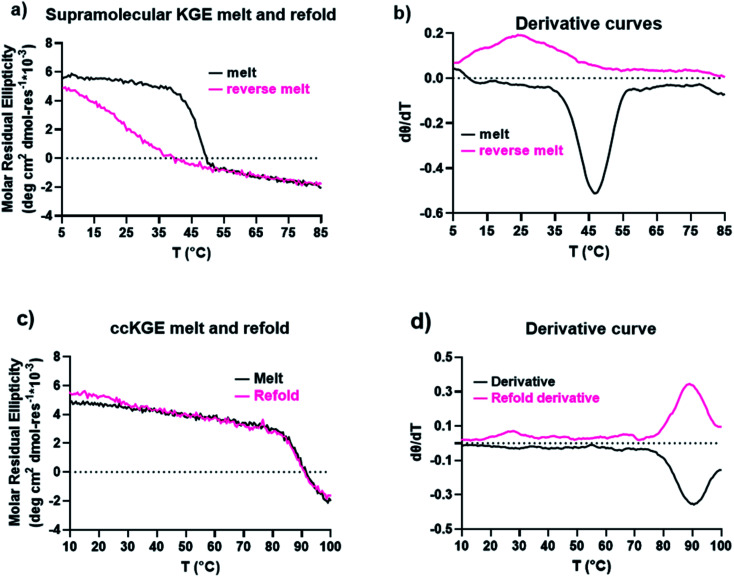
CD results of KGE triple helices. (a) CD melt and refold curves of supramolecular KGE triple helix. (b) The first-order derivative curves of the melting curves in (a). (c) CD melting and refolding curves of covalently captured KGE triple helices from benzyl protecting group removal. (d) The first-order derivative curves of the melting curves in (c).

We characterized the thermal stability and refolding of the supramolecular and covalently captured triple helices. The melting temperature of the supramolecular KGE triple helices is 46 °C (Fig. 5a and b). The refolding curve of the supramolecular KGE triple helix shows the increase of molar residual ellipticity at 225 nm as the temperature decreases, indicating triple helix reformation, but the refolding curve shows significant hysteresis compared to unfolding indicative of the slow folding typical of PP2 triple helices (Fig. 5a). We then characterized the covalently captured samples after protecting group removal (Fig. 5c and d). The melting temperature of the covalently captured KGE triple helix was found to be a remarkable 90 °C. Compared to the supramolecular trimer, covalently capping the end of this triple helix resulted in a 44 °C increase of its thermal stability. The refolding experiment of the covalently captured sample shows nearly superimposed refolding and the melting curves suggestive of the rapid refolding enforced by the isopeptide amide bonds (in all the refolding studies some evaporation of solvent, up to 8% by volume, was seen by the end of the 17 hour study. This results in slightly increased observed MRE values during refolding).

### Application the selective covalent capture technique to peptides derived from SP-A

The selected sequence in the collagenous domain of SP-A has four internal salt–bridge interactions which involves one lysine, one aspartate and two glutamate residues. Depending on the registration, the lysine residue can potentially make contact with each of the negatively charged residues through axial or lateral interactions. Therefore, the minimal side chain protection strategy suggests protection of lysine with ivDde (Scheme 1) while leaving the negatively charged residues unprotected. We synthesized two variations of the SP-A peptide for covalent capture: one with covalent capture sequences at only the N-terminus and one with covalent capture sequences at both the N- and C-terminus. The self-assembly and covalent capture of SP-A1 peptides (Table S1† and Scheme 1C) will result in the formation of three covalent bonds at the N-terminus of the triple helix. That of SP-A2 peptide (Table S1† and Scheme 1D) will result in six covalent bonds formed, three bonds at each terminus of the triple helix. We consider the covalent bond formation at only one end of the triple helix may allow more flexibility for binding molecules to access the internal binding sites while the double covalent capture provides additional stability and increased rate of folding. Depending on the application, either approach may be beneficial.

**Fig. 6:**
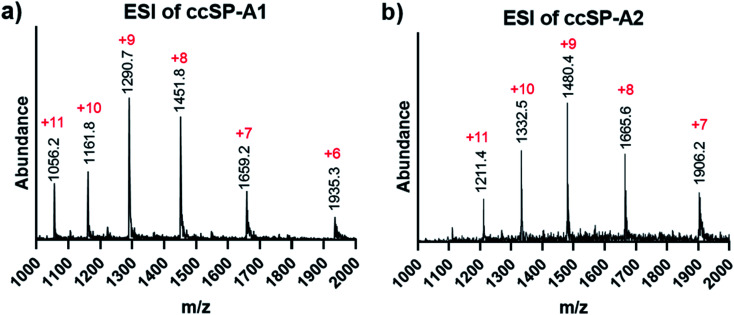
ESI mass spectra of covalently captured SP-A branch triple helices. (a) Covalently captured SP-A1 triple helix after ivDde deprotection. (b) Covalently captured SP-A2 triple helix after ivDe deprotection.

Both ivDde-protected peptides SP-A1 and SP-A2 folded into triple helices according to the CD results (Fig. S16 and S17†). We then carried out the covalent capture experiment as detailed in the ESI.† Covalent capture of natural collagen mimetic peptides with multiple charged amino acid residues can be problematic according to our observation. Precipitation appeared in the preliminary trials which can be caused by the formation of kinetic assemblies with undesired registration. To avoid this, the defence collagen peptides were equilibrated longer for self-assembly and covalently captured longer for complete reaction. After the ivDde protecting group removal, we were able to purify and obtain the selectively covalently captured triple helix according to the mass spectra (Fig. 6 and Table S1†).

**Fig. 7:**
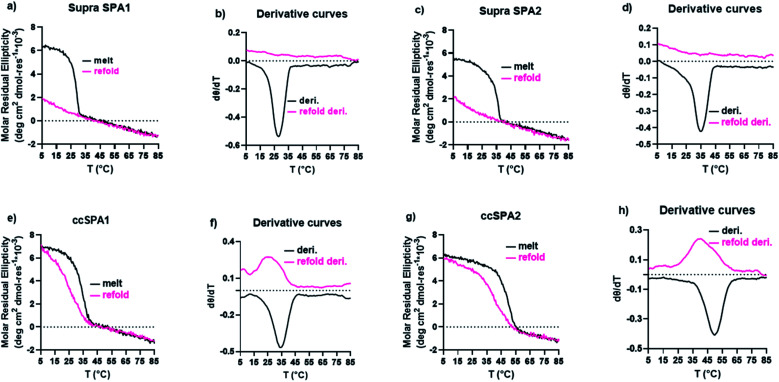
CD results of SP-A triple helices. (a) CD melt and refolding curves of supramolecular SP-A1 triple helices. (b) first-order derivative of the melting curves in part (a). (c) CD melt and refolding curves of supramolecular SP-A2 triple helices. (d) first-order derivative of the melting curves in part (d). (e) CD melt and refolding curves of covalently captured SP-A1 triple helix. (f) first-order derivative of the melting curves in part *e.g.* CD melt and refolding curves of covalently captured SP-A2 triple helix. (h) first-order derivative of the melting curves in part (g).

The supramolecular SP-A1 and SP-A2 triple helices have a melting temperature of 28 and 35 °C, respectively (Fig. 7a–d). The SP-A2 triple helix differs from the SP-A1 triple helix by having an extra PKGEOG sequence at the C-terminus (Table S1†). Like the simple supramolecular KGE triple helix, for both SP-A1 and SP-A2, the refolding curves show the hysteresis between the melting curve and the refolding curve and the maximum MRE value was not obtained during the refolding period (full refolding is achieved after days) demonstrating the slow folding kinetics.

We prepared ccSP-A1 by covalently capturing the N-terminus of the trimer followed by deprotection of lysine. The melting temperature of ccSP-A1 is 35 °C, which is 7 °C higher than its supramolecular counterpart (Fig. 7e and f). The refolding curve of the covalently captured ccSP-A1 triple helix shows some hysteresis from the melting curve, but is less prominent compared to the supramolecular version. ccSP-A2 melting temperature was found to be 50 °C which is 15 °C higher than the supramolecular counterpart. The reverse melt curve shows minor hysteresis from the melting curve, but it is less prominent compared to either the supramolecular SP-A2 trimer or the ccSP-A1 trimer that is covalently captured at only one end. Compared to the template peptides ccKGE, however, the refolding of ccSP-A2 still shows some hysteresis. We believe this is due to the complex natural amino acid sequences of SP-A which are known to have much slower folding kinetics compared to simpler (POG)n sequences. For example, the supramolecular SP-A peptides fold much slower than the supramolecular KGE peptides and the folded triple helices have lower thermal stability. We can conclude that, covalent capture of triple helices increased the thermal stability of the collagen-like domain from protein SP-A. These natural sequences have very slow folding kinetics, but covalent capture technique effectively increased the folding speed and thermal stability. The improvements are more prominent when both ends of the trimer are covalently captured.

## Conclusions

In this work we demonstrated a minimal protecting strategy for the covalent capture study of collagen triple helices. Our approach allows the covalent stabilization of triple helices with nearly any sequence. This strategy does not appreciably affect collagen triple helix conformation nor solubility. The deprotection conditions studied here avoid the use of organic reagents, instead removing the protecting group easily under aqueous condition. The methods developed here were tested with natural sequences from SP-A which cannot be covalently captured without protecting groups. The covalent triple helix was found to refold more rapidly and have a thermal stabilities 7 °C or 15 °C higher than their supramolecular analogues depending of if a single or double covalent capture strategy was employed. Perhaps most important for applications of triple helices, either approach allows their use in situations where very low concentrations must be used. The selective covalent capture methods described here provide a blueprint for successful stabilization of virtually any collagen triple helix which should expand their use in a large variety of biological applications.

## Data availability

The datasets supporting this article have been uploaded as part of the ESI.†

## Author contributions

Jeffrey D. Hartgerink contributed to the conceptualization of the research. Le Tracy Yu contributed to experimental investigation of the work. Jeffrey D. Hartgerink and Le Tracy Yu both contributed to the experimental designs and writing of the manuscript.

## Conflicts of interest

There are no conflicts to declare.

## Supplementary Material

SC-013-D1SC06361H-s001
