# Supplementary material for: Selective covalent capture of collagen triple helices with a minimal protecting group strategy
Source: Chem Sci. 2022 Feb 17;13(9):2789–96. doi: 10.1039/d1sc06361h (PMC8890135; doi:10.1039/d1sc06361h)
Supplement: SC-013-D1SC06361H-s001 [file SC-013-D1SC06361H-s001.pdf]

Electronic Supplementary Material (ESI) for Chemical Science.
This journal is © The Royal Society of Chemistry 2022

Supplementary Information

Selective Covalent Capture of Collagen Triple Helices with Minimal Protecting Group Strategy

Le Tracy Yu, Jeffrey D. Hartgerink*

Rice University, Department of Chemistry and Department of Bioengineering, Houston, TX 77005.

KEYWORDS: Collagen, Peptide, Covalent Capture, ivDde, OBzl, OAll, ODmab, Deprotection.

Contents

1. Peptide Synthesis 1
   1.1 Reagents and Materials 1
   1.2 Synthesis Procedures 1
2. Peptide Purification and Characterization 1
   2.1 Peptide Purification 1
   2.2 Mass spectrometry 1
3. Covalent Capture 7
4. Deprotection of the Side Chain Protecting Groups on Internal Charged Amino Acid Residues 7
   4.1. Deprotection of ivDde protecting group (Condition 1 and 8 in the main text) 7
   4.2 Deprotection of allyl ester protecting group (Condition 2, 9, 10) 7
   4.3 Deprotection of benzyl ester protecting group (Condition 3, 4, 5, 11, 12) 8
   4.4 Deprotection of Dmab protecting group (Condition 6,7, 13, 14) 8
   4.5 UPLC traces of OAll and OBzl removal at different conditions 8
5. Circular Dichroism 9
References 11

1. Peptide Synthesis

1.1 Reagents and Materials

Reagents for peptide synthesis. Rink Amide MBHA resin LL (100-200 mesh was purchased from EMD Millipore. Amino acids, N,N-Diisopropylethylamine (DIEA), ninhydrin, tetrachloro-1,4-benzoquinone (chloranil), trifluoroacetic acid (TFA), anisole, triisopropylsilane (TIPS) were purchased from Sigma Aldrich. N,N-dimethylformamide (DMF), dichloromethane (DCM), ethane dithiol (EDT) were purchased from Fisher Scientific. Activating reagent 2-(1H7-azabenzotriazol-1-yl)-1,1,3,3-tetramethyl uronium hexafluorophosphate methanaminium (HATU) was purchased from P3bio.

Reagents for peptide purification. Acetonitrile (HPLC grade) was purchased from Fischer Scientific. Materials for mass spectrometry α-Cyano-4-hydroxycinnamic acid (CHCA) was purchased from Sigma Aldrich; acetonitrile was purchased from Fischer Scientific.

Reagents and materials for sample preparation and reactions. MES hemisodium salt, 1-hydroxybenzotriazole hydrate (HOBt) ≥97.0% (T), N-(3-Dimethylaminopropyl)-N'-ethylcarbodiimide hydrochloride (EDC), sodium carbonate (K_2CO_3), were purchased from Sigma Aldrich. Hydrazine hydrate, 100% (Hydrazine, 64%) was purchased from Fisher Scientific. Pierce™ Protein Concentrators PES, 3K MWCO was purchased from Fischer Scientific.

1.2 Synthesis Procedures

Amino acid coupling. All the peptides were synthesized manually using solid phase peptide synthesis with Fmoc chemistry. All the peptides are N-terminally acetylated and C-terminally amidated to avoid terminal charge repulsion. In detail, a low loading Rink Amide Resin (0.34 mmol/gram) was used. The resin was swelled twice for 1 min with dichloromethane (DCM), 10 mL for each (2*10 mL). Then the resin was washed with N,N-dimethylformamide (DMF) twice for 1 min with 10 mL of DMF for each time (2*10 mL). After that, 10 mL of 25% (v.v) solution of piperidine in DMF was added into the resin to deprotect the Fmoc protecting group; deprotection has two cycles and was 5 mins long for each cycle. After deprotection, the resin was washed 5 times with DMF, 10 mL for each time (5*10 mL). Ninhydrin test (Kaiser Test) and chloranil test were then utilized to detect the presence of primary amine and secondary amine, respectively. The amino acid, activating reagent 2-(1H7-azabenzotriazol-1-yl)-1,1,3,3-tetramethyl uronium hexafluorophosphate methanaminium (HATU) and a weak base diisopropylethylamine (DiEA) were dissolved in 10 mL of DMF and mixed for 1 min for pre-activation during which the reaction solution turned light yellow. The molar ratio of the reagents in each coupling was 1:4:4:6 (resin/amino acid/HATU/DiEA); for the coupling cycle containing ^{15}N-labeled glycine, the ratio was halved to be 1:2:2:3. After pre-activation, the reaction solution was added to the resin and reacted in a constantly agitated reaction vessel till completion. Repeat ninhydrin or chloranil test after coupling. Procedures for deprotection, coupling as well as tests were repeated for each cycle of amino acid attachment.

Peptide N-terminal Acetylation. After coupling all the amino acid residues and the final cycle of deprotection, peptide was acetylated with acetic anhydride. The molar ratio of the acetylation reagents was 1:8:4 (resin/acetic anhydride/DiEA). The acetylation reagents were mixed and added into the DCM-washed resin and reacted for 45 mins. Acetylation was repeated once more before cleavage. After acetylation, the resin was washed 3 times with DCM, 10 mL for each (3*10 mL) and continued with cleavage. Ninhydrin or chloranil test was carried out to confirm complete acetylation.

Peptide Cleavage. The peptide on resin was mixed with the cleavage cocktail (90% TFA/2.5% Anisole/2.5% Milli Q H_2O/2.5% TIPS/2.5% EDT by volume) and reacted for 3 hours. The cleaved peptide was then drained into a clean r.t flask and half the amount of cleavage cocktail was added to wash the resin; all the solution was collected into the flask. The TFA was blown off with nitrogen gas. Approximately 50 mL of ice-cold diethyl ether was added into the peptide solution to triturate the peptide. The mixture was then centrifuged to obtain the white pellet of the crude peptide. The white pellet was washed once more with 30 mL of cold ether to further remove the cleavage cocktail. After that, the crude peptide was covered with Kim wipes and left to dry in the fume hood. The acid labile side chain protecting groups were removed during cleavage. The side chains of the internal lysine, aspartic acid and glutamic acid were orthogonally protected and were removed after successful covalent capture.

2. Peptide Purification and Characterization

2.1 Peptide Purification

The dried crude peptide was dissolved in milli Q H_2O to a concentration of approximately 18 mg/mL. The peptide solution was purified with reversed-phase HPLC (Varian PrepStar220) using a preparative C-18 column. The mobile phase is acetonitrile with 0.05% TFA by volume (solvent B) vs milli Q H_2O with 0.05% TFA by volume (solvent A). The void time of the system is 2.50 mins. The collected fractions of purified peptides were rotovapored of the acetonitrile and frozen and then lyophilized to remove the moisture. The purified peptides were reinjected into the UPLC to confirm the purity. The UPLC gradient was 0.75 min-6.75 min, 5-35% solvent B.

2.2 Mass spectrometry

Bruker AutoFlex Speed MALDI ToF and Bruker MicroToF ESI LC-MS System were used to characterize the mass-to-charge ratio of ions of peptides. For MALDI spectrometry measurement, 1.5 uL of 10 mg/mL matrix CHCA in 50% (v.v.) MQ H_2O, 50% (v.v) acetonitrile and 0.1% (v.v) TFA was mixed with 1.5 ul 20 mg/mL peptide solutions for sample co-crystallization. For LC-MS measurement, samples were prepared into a 0.5 mg/mL concentration in MQ H_2O. The measured and the expected mass-to-charge ratio was listed in Table S1.

Table S1. Peptide Molecular Weight Information

Peptide	Sequence	Expected [M+H]+	Observed [M+H]+
KGE	PKGEOGPOGPOGPKGEOGPOGPOGPKGEOG	2873.05	2873.11
KGE	PKGEOGPOGPOGPK(ivDde)GEOGPOGPOGPKGEOG	3079.33	3079.63
KGE(OAll)	PKGEOGPOGPOGPKGE(OAllyl)OGPOGPOGPKGEOG	2912.41	2912.37
KGE(ODmab)	PKGEOGPOGPOGPKGE(Dmab)OGPOGPOGPKGEOG	3184.65	3184.43
KGE(OBzl)	PKGEOGPOGPOGPKGE(OBzl)OGPOGPOGPKGEOG	2963.33	2963.48
SP-A1	MOGLOGRDGLOGAOGAOGERGDKGEOGERGLOG	3886.44	3887.13
SP-A2	PKGEOGMOGLOGRDGLOGAOGAOGERGDGEOGERGLOGPKGEOG	4469.75	4469.2
SP-A1_K	PKGEOGMOGLOGRDGLOGAOGAOGERGDK(ivDde)GEOGERGLOG	4092.42	4092.12
SP-A2_K	PKGEOGMOGLOGRDGLOGAOGAOGERGDK(ivDde)GEOGERGLOGPKGEOG	4675.04	4675.4
ccK(ivDde)GE	PKGEOGPOGPOGPKGEOGPOGPOGPKGEOG<br>PKGEOGPOGPOGPKGEOGPOGPOGPKGEOG<br>PKGEOGPOGPOGPKGEOGPOGPOGPKGEOG	9131.99	9132.37
ccKGE(OAll)	PKGEOGPOGPOGPKGEOGPOGPOGPKGEOG<br>PKGEOGPOGPOGPKGEOGPOGPOGPKGEOG<br>PKGEOGPOGPOGPKGEOGPOGPOGPKGEOG	8629.23	8628.22
ccKGE(OBzl)	PKGEOGPOGPOGPKGEOGPOGPOGPKGEOG<br>PKGEOGPOGPOGPKGEOGPOGPOGPKGEOG<br>PKGEOGPOGPOGPKGEOGPOGPOGPKGEOG	8781.99	8804.65<br>[M+Na]+
ccSP-A1_K	PKGEOGMOGLOGRDGLOGAOGAOGERGDKGEOGERGLOG<br>PKGEOGMOGLOGRDGLOGAOGAOGERGDKGEOGERGLOG<br>PKGEOGMOGLOGRDGLOGAOGAOGERGDKGEOGERGLOG	12203.26	12225.8<br>[M+Na]+
ccSP-A2_K	PKGEOGMOGLOGRDGLOGAOGAOGERGDKGEOGERGLOGPKGEOG<br>PKGEOGMOGLOGRDGLOGAOGAOGERGDKGEOGERGLOGPKGEOG<br>PKGEOGMOGLOGRDGLOGAOGAOGERGDKGEOGERGLOGPKGEOG	13917.12	13953.8<br>[M+K]+
ccKGE from ivDde removal	PKGEOGPOGPOGPKGEOGPOGPOGPKGEOG<br>PKGEOGPOGPOGPKGEOGPOGPOGPKGEOG<br>PKGEOGPOGPOGPKGEOGPOGPOGPKGEOG	8511.45	8511.4
ccKGE from Allyl removal	PKGEOGPOGPOGPKGEOGPOGPOGPKGEOG<br>PKGEOGPOGPOGPKGEOGPOGPOGPKGEOG<br>PKGEOGPOGPOGPKGEOGPOGPOGPKGEOG	8511.45	8511.4
ccKGE from Dmab removal	PKGEOGPOGPOGPKGEOGPOGPOGPKGEOG<br>PKGEOGPOGPOGPKGEOGPOGPOGPKGEOG<br>PKGEOGPOGPOGPKGEOGPOGPOGPKGEOG	8511.45	8477.84<br>(extra $2H_2O$ losses)
ccKGE from Bzl removal	PKGEOGPOGPOGPKGEOGPOGPOGPKGEOG<br>PKGEOGPOGPOGPKGEOGPOGPOGPKGEOG<br>PKGEOGPOGPOGPKGEOGPOGPOGPKGEOG	8511.45	8511.6
ccSP-A1 from ivDde removal	PKGEOGMOGLOGRDGLOGAOGAOGERGDKGEOGERGLOG<br>PKGEOGMOGLOGRDGLOGAOGAOGERGDKGEOGERGLOG<br>PKGEOGMOGLOGRDGLOGAOGAOGERGDKGEOGERGLOG	11607.32	11608.3
ccSP-A2 from ivDde removal	PKGEOGMOGLOGRDGLOGAOGAOGERGDKGEOGERGLOGPKGEOG<br>PKGEOGMOGLOGRDGLOGAOGAOGERGDKGEOGERGLOGPKGEOG<br>PKGEOGMOGLOGRDGLOGAOGAOGERGDKGEOGERGLOGPKGEOG	13302.25	13325.0<br>[M+Na]+

*"cc" represents covalent capture. The covalent capture samples were removed of the respective protecting groups. The spectra comparison of trimers before and after covalent capture are listed below.

KGE

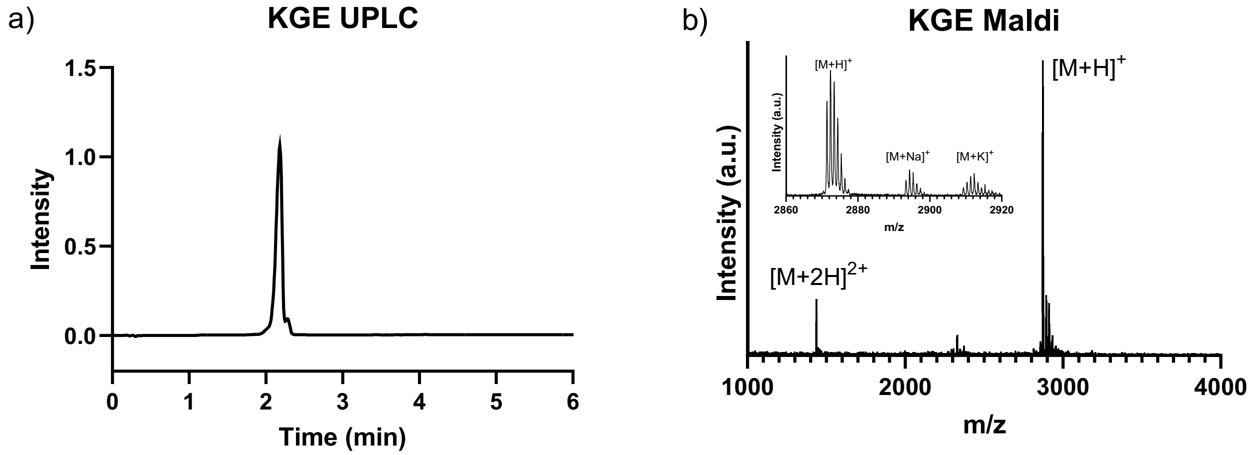

Figure S1. a) UPLC trace of purified peptide KGE. The gradient of solvent B is 0.75-6.75 min 5-35% B. b) MALDI mass spectrum of purified peptide KGE.

K(ivDde)GE

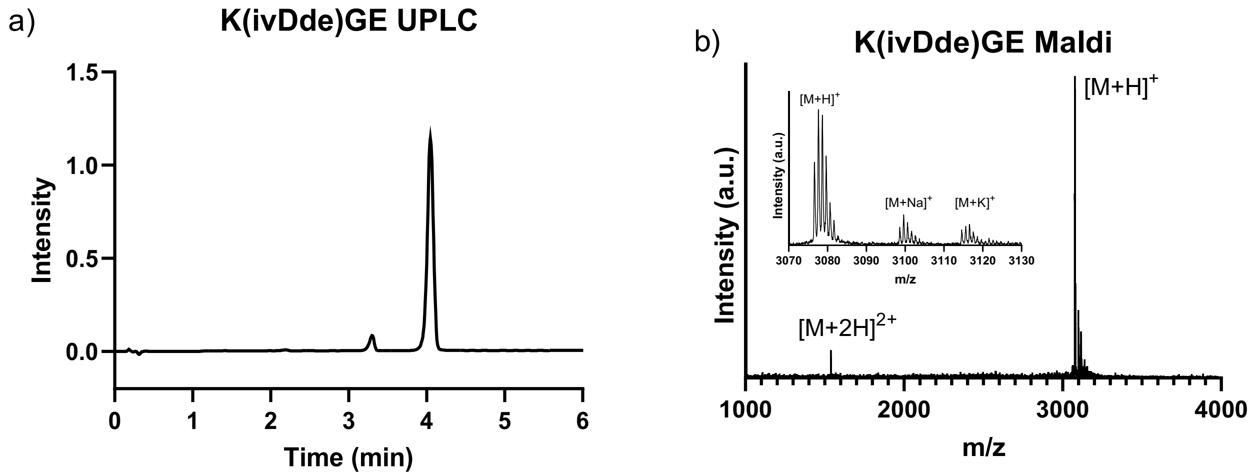

Figure S2. a) UPLC trace of purified peptide K(ivDde)GE. The gradient of solvent B is 0.75-6.75 min 5-35% B. b) MALDI mass spectrum of purified peptide K(ivDde)GE.

KGE(OAll)

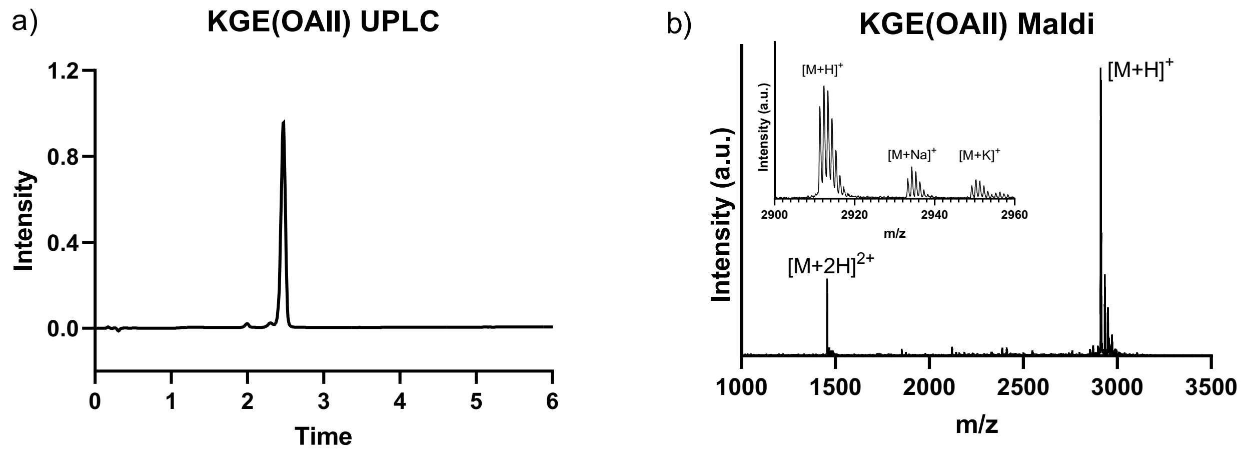

Figure S3. a) UPLC trace of purified peptide KGE(OAll). The gradient of solvent B is 0.75-6.75 min 5-35% B. b) MALDI mass spectrum of purified peptide KGE(OAll).

KGE(OBzl)

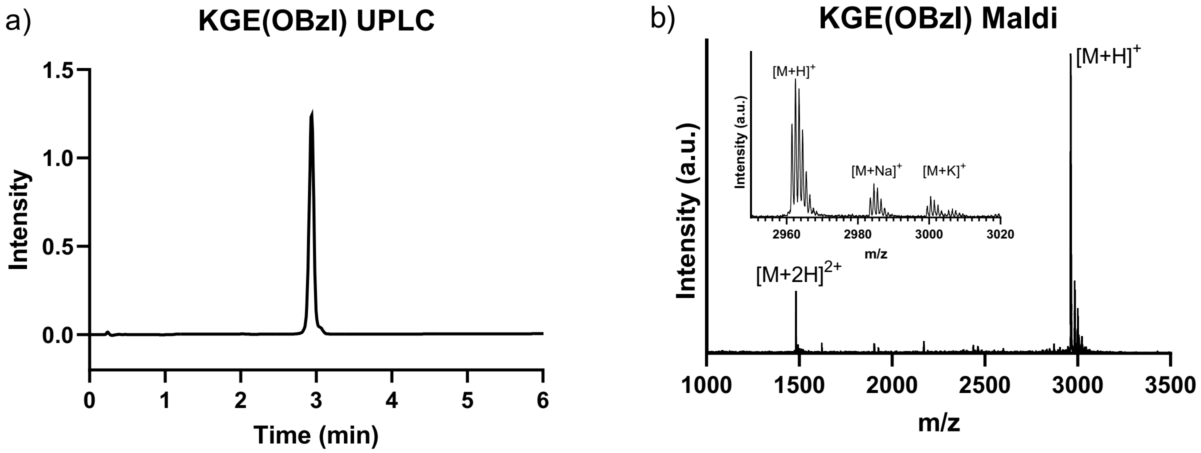

Figure S4. a) UPLC trace of purified peptide KGE(OBzl). The gradient of solvent B is 0.75-6.75 min 5-35% B. b) MALDI mass spectrum of purified peptide KGE(OBzl).

KGE(ODmab)

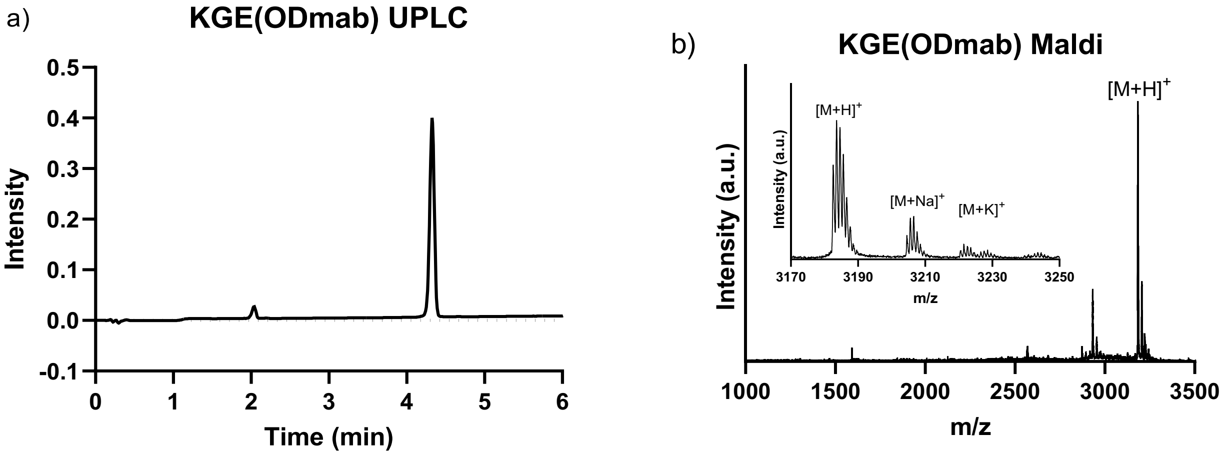

Figure S5. a) UPLC trace of purified peptide KGE(ODmabl). The gradient of solvent B is 0.75-6.75 min 5-35% B. b) MALDI mass spectrum of purified peptide KGE(ODmab).

SP-A_1

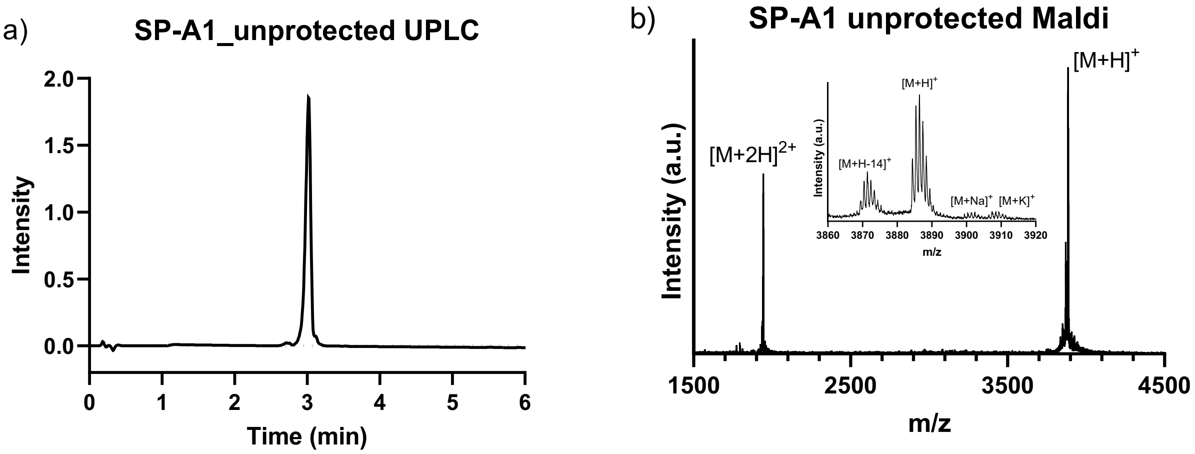

Figure S6. a) UPLC trace of purified peptide SP-A1. The gradient of solvent B is 0.75-6.75 min 5-35% B. b) MALDI mass spectrum of purified peptide SP-A1.

SP-A1_K

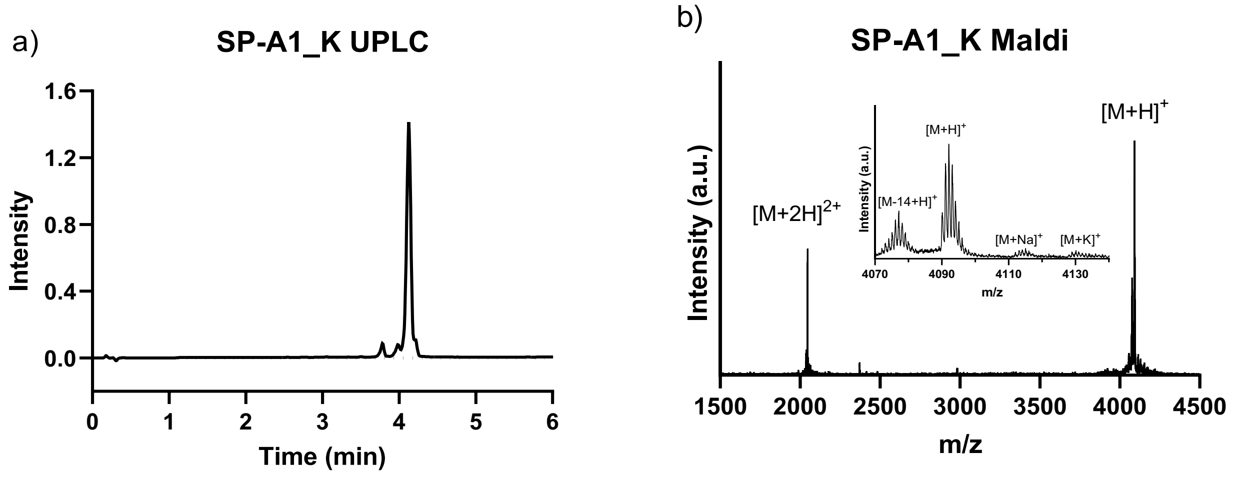

Figure S7. a) UPLC trace of purified peptide SP-A1_K. The gradient of solvent B is 0.75-6.75 min 5-35% B. b) MALDI mass spectrum of purified peptide SP-A1_K.

SP-A2

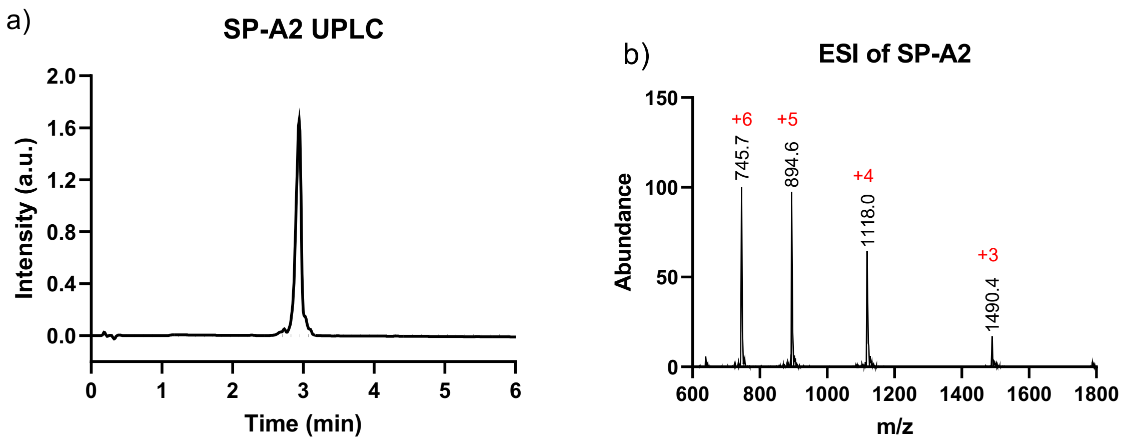

Figure S8. a) UPLC trace of purified peptide SP-A2. The gradient of solvent B is 0.75-6.75 min 5-35% B. b) ESI mass spectrum of purified peptide SP-A2.

SP-A2_K

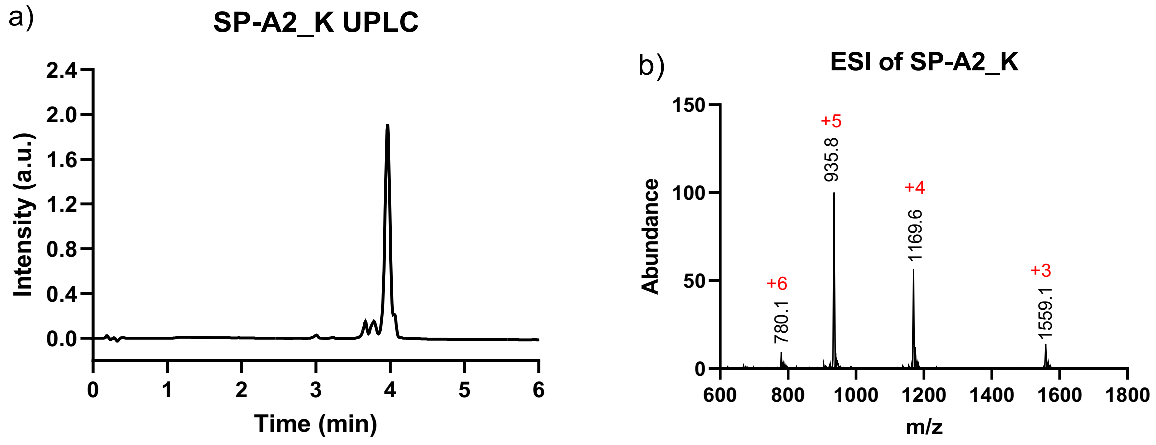

Figure S9. a) UPLC trace of purified peptide SP-A2_ K. The gradient of solvent B is 0.75-6.75 min 5-35% B. b) ESI mass spectrum of purified peptide SP-A2_K.

Maldi of ccK(ivDde)GE, ccKGE(OAll), and ccKGE(OBzl)

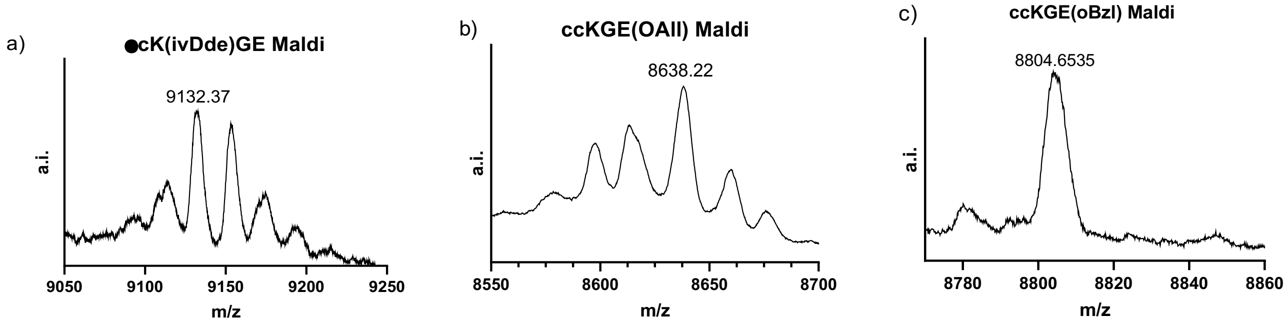

Figure S10. Maldi mass spectrum of covalently captured triple helices with a) ivDde protecting groups, b) OAll protecting groups and c) OBzl protecting groups. Triple helices samples are pre-purification and pre-deprotecting.

ESI mass spec of ccSP-A1_K and ccSP-A2_K

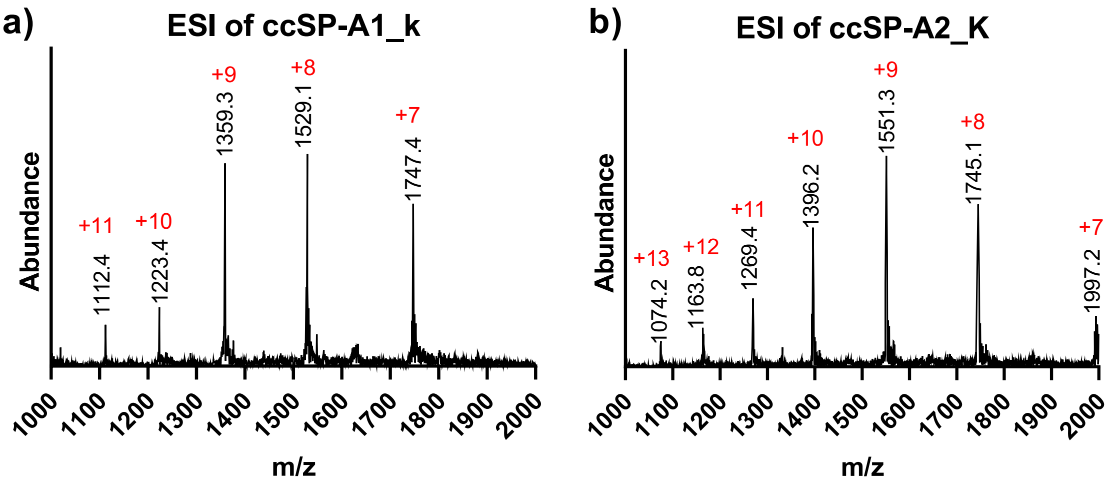

Figure S11. ESI mass spectra of covalently captured SP-A triple helices with ivDde protecting groups. a) ccSP-A1 _K b) ccSP-A2 _K.

UPLC traces of ccSP-A1 and ccSP-A2

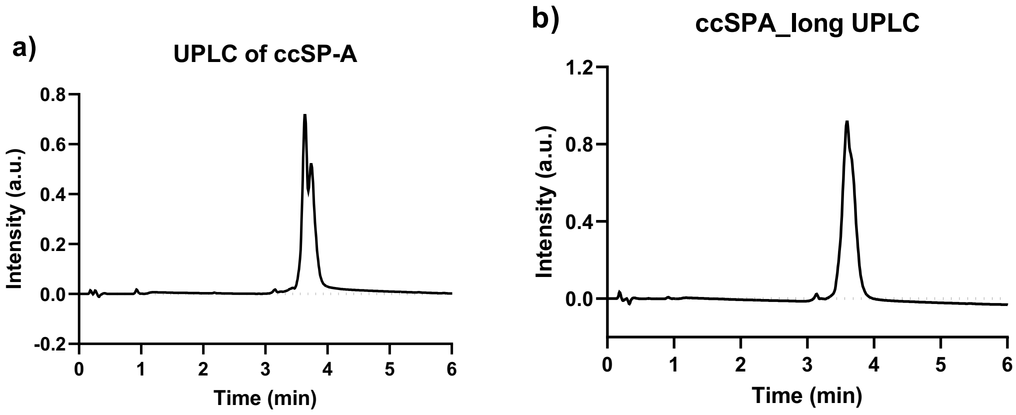

Figure S12. UPLC traces of purified covalently captured triple helices. The gradient is 5-35% B 0.5-6.5 min. a) ccSP-A1 from the covalent capture of SP-A1_K triple helix followed by the ivDde protecting group removal. b) ccSP-A2 from the covalent capture of SP-A2_K triple helix followed by the ivDde removal.

3. Covalent Capture

The covalent capture procedures were referred to the literature.[1] In detail, HPLC purified peptide was dissolved in 100 mM MES buffer pH 6.1 to result in a 3 mM peptide concentration. The pure peptide content was approximately 76% according to our previous analysis. 12 mM Hydroxybenzotriazole and 120 mM 1-ethyl-3-(3-dimethylaminopropyl) carbodiimide (EDC) solutions were prepared in 100 mM MES buffer. After the monomer peptide folded into collagen triple helices and the thermal stability was confirmed with circular dichroism, the peptide solution was mixed with EDC and HOBt solutions in a 1:1:1 volume ratio. The resulted solution has the peptide concentration of 1 mM in 4 mM HOBt and 40 mM EDC. The mixture was kept at 5 °C and was vortexed every hour at the first 4 hours of covalent capture reaction. After 4 days of covalent capture reaction, the reaction was quenched with different conditions (described below) and continued with removal of the side chain protection groups of the internal charged amino acid residues. EDC and HOBt need to be removed with Spin column, HPLC, or quenching reagents before sample characterization with mass spectrometry.

4. Deprotection of the Side Chain Protecting Groups on Internal Charged Amino Acid Residues

Scheme S1. Reaction Scheme of the Selective Covalent Capture using ivDde, OBzl and OAllyl Protecting Group

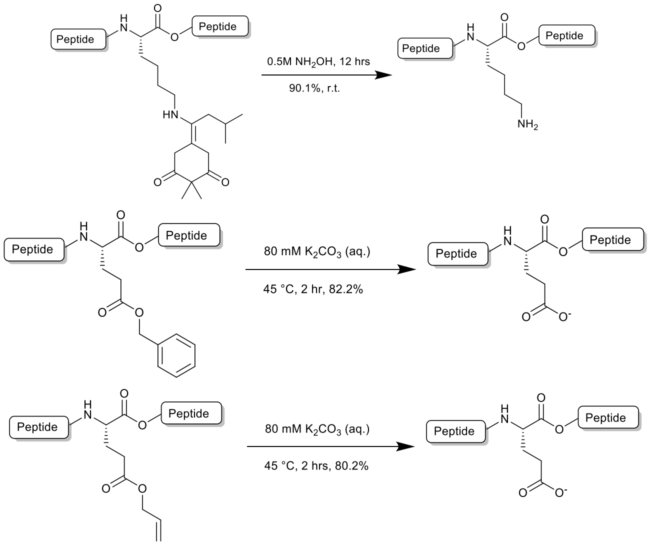

Scheme S1. Successful deprotection condition of template triple helices. Before the deprotection, the reaction solution was treated with spin column (MWCO 3K) to remove part of EDC and HOBt.

4.1. Deprotection of ivDde protecting group (Condition 1 and 8 in the main text)

Condition 1. Removal of ivDde from monomer peptide. The reaction works for either crude peptide or purified peptide. The monomer peptide was dissolved into 100 mM MES buffer till a concentration of approximately 10 mg/mL. Add equal volume of 1M hydroxyamine (NH_2OH). Mix and agitate the reaction solution overnight at room temperature. Then quench hydroxyamine with 1M HCl till the solution is acidic.

Condition 8. Removal of ivDde from covalently captured trimer. After covalent capture, we used the Pierce™ Protein Concentrators PES, 3K MWCO, 0.5 mL to remove EDC and HOBt to avoid extra iso-peptide bond formation after the deprotection. The sample solution was centrifuged three times, each at 12 000 rcf for 30 mins. If uses 3K MWCO, 2-6 mL concentrator, the samples were treated at 4700 rcf for 30 mins for 3 time. Between each centrifugation, certain volume of 100 mM MES buffer was added into the sample tube to bring the volume of solution to the starting point. After centrifugation, collect the sample solution and equal volume of 1M NH_2OH was added into the solution for ivDde removal. The solution was mixed, reacted, and quenched as described in Condition 1.

4.2 Deprotection of allyl ester protecting group (Condition 2, 9, 10)

Condition 2. Removal of allyl easter from monomer peptide. The reaction conditions refer to literature with modification.[2] According to our trials, the condition works only for purified peptide. The peptide was prepared into approximately 10 mg/mL solution in 50 mM phosphate buffer (pH 7). 25 eq. of $[Pd(All)Cl]_2$ catalyst and 25 eq. of L-Glutathione reduced (GSH) were added into the solution. The pH was adjusted to be 8 with 1M NaOH. The solution was mixed well and agitated at 37 °C for 30 mins. To quench

the reaction, 40 eq. dithiothreitol (DTT) was added. The catalyst then precipitated, and the sample solution was centrifuged three times to remove the catalyst. The reaction solution was purified with HPLC.

Condition 9. The covalently captured reaction solution was pretreated with Pierce™ Protein Concentrators PES, 3K MWCO as described previously. Similarly, 25 eq. of $[Pd(All)Cl]_2$ catalyst and 25 eq. of L-Glutathione reduced (GSH) were added into the solution. The pH was adjusted to be 8 with 1M NaOH. The solution was mixed well and agitated at 45 °C for > 12 hrs. After quenching the reaction with DTT, peptide was purified and characterized. We observed incomplete deprotection.

Condition 10. The covalently captured reaction solution was pretreated with Pierce™ Protein Concentrators PES, 3K MWCO as described previously. Certain amount of 1M K_2CO_3 was added into the solution till the concentration of K_2CO_3 was approximately 80 mM. The pH was confirmed to be approximately 11. The solution was agitated at 45 °C for 2 hrs. The reaction was quenched with 1M HCl till the solution was acidic.

4.3 Deprotection of benzyl ester protecting group (Condition 3, 4, 5, 11, 12)

Condition 3. Removal of benzyl ester from peptide monomers with Pd black. Dissolve the peptide in glacier acetic acid till a concentration of 10 mg/mL. Add Pd black into the solution. Purge the reaction solution three times with N_2 and vacuum. Add a H_2 balloon into the reaction solution. Agitate the reaction solution and follow the reaction progress with UPLC. To quench the reaction, centrifuge the solution three times to get rid of Pd catalyst.

Condition 4 and 5. Removal of benzyl ester from the monomer peptide with saponification. These conditions work for either purified or crude peptide solutions. Dissolve the peptide in MQ H_2O till a concentration of 10 mg/mL. In condition 4, add 1M NaOH till pH close to 11; in condition 5, add 1M K_2CO_3 till the pH close to 11. Mix and agitate the reaction solution at 45 °C for 1 hr. Add 1M HCl to quench the reaction till the solution is acidic.

Condition 11 and 12. Removal of benzyl ester from the covalently captured trimer. The samples need to be pretreated with Pierce™ Protein Concentrators PES, 3K MWCO as described above. The deprotection and workup procedures are as described in conditions 4 and 5.

4.4 Deprotection of Dmab protecting group (Condition 6,7, 13, 14)

Condition 6 and 7. Removal of Dmab protecting group from monomer peptides. Purified peptides were dissolved into 10 mg/mL concentration in MQ H2O. For condition 6, 2% wt. hydrazine was added. For condition7, equal volume of 1M NH_2OH was added into the solution. The reaction solutions were mixed and reacted at r.t. To quench the reaction, 1M HCl was added till the solution was acidic. Our data suggest the hydrazine condition can realize almost quantitative conversion within 30 mins and that was 2 hrs for hydroxyamine condition. No side product was observed.

Condition 13 and 14. Removal of Dmab protecting group from the covalently captured trimer. The covalently captured trimer solution was pretreated with Pierce™ Protein Concentrators PES, 3K MWCO as described above. In condition 13, 2% wt. hydrazine was added into the solution and in condition 14, equal volume of 1M hydroxyamine was added. The reaction was mixed and agitated at r.t. for 2 hrs in condition 13 and 6hrs in condition 14. We observed the cyclized side product as the major species.

4.5 UPLC traces of OAll and OBzl removal at different conditions

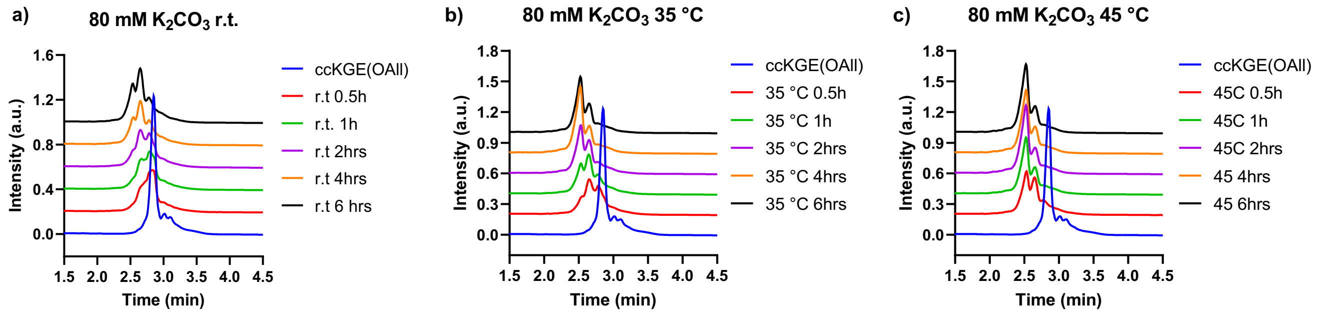

Figure S13. UPLC traces of OAll removal from covalently captured triple helix ccKGE(Obzl). The deprotection was carried out at in 80 mM aqueous K_2CO_3 solutions at a) room temperature, b) 35 °C and c) 45 °C. The percentage of expected product was calculated by integrating the product peak area eluted at 2.6 min.

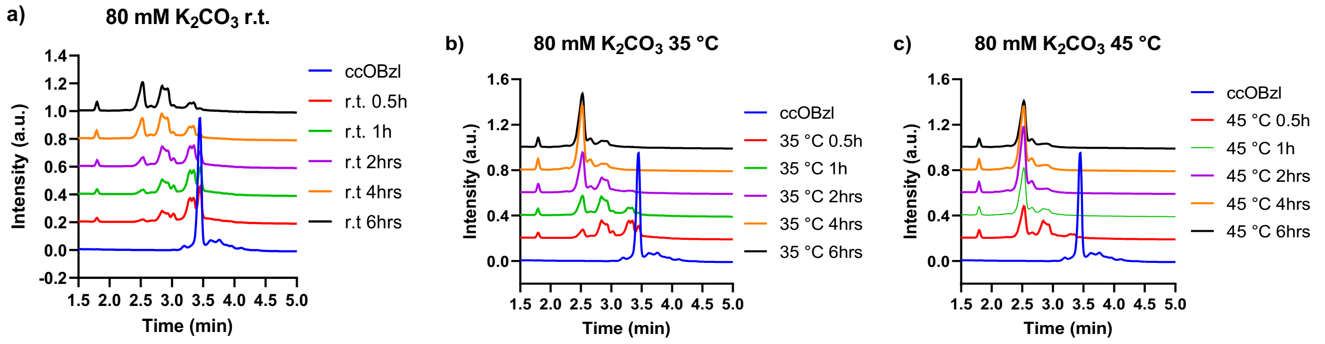

Figure S14. UPLC traces of OBzl removal from covalently captured triple helix ccKGE(Obzl). The deprotection was carried out at in 80 mM aqueous K_2CO_3 solutions at a) room temperature, b) 35 °C and c) 45 °C. The percentage of expected product was calculated by integrating the product peak area eluted at 2.5 min.

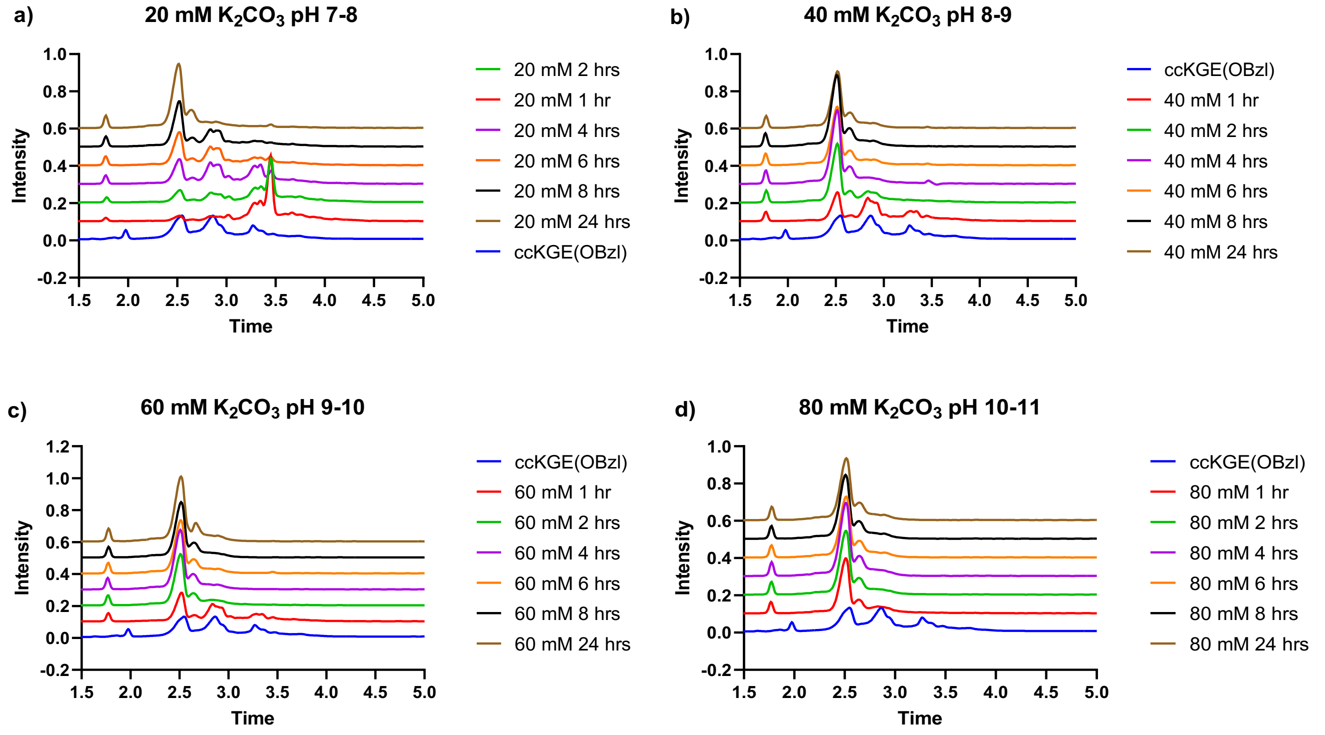

Figure S15. UPLC traces of OBzl removal from covalently captured triple helix ccKGE(Obzl). The deprotection was carried out at 45 °C in a) 20 mM b) 40 mM c) 60 mM and d) 80 mM aqueous K_2CO_3 solutions. The percentage of expected product was calculated by integrating the product peak area eluted at 2.5 min.

5. Circular Dichroism

The circular dichroism (CD) data were collected on a Jasco J-810 spectropolarimeter equipped with a Peltier temperature controller. Spectrum measurement was collected at 5 °C. The spectra scanning used 1 mm cuvette and a peptide concentration of 0.03 mM. The melting curves were collected from 5 °C to 85/95 °C with a heating rate of 10 °C/hour at the wavelength that gives the maximum MRE value of each sample. The melting data was collected using 1 mm cuvette with a peptide concentration of 0.3 mM to obtain better signal. For the refolding experiment, the sample was kept at 85/95 °C for 30 mins to fully denature the folded triple helices and then the temperature was cooled down to 5 °C with a cooling rate of 10°C/hour. In some cases, the volume of the sample solution decreased from 200 uL to approximately 185 uL by the completion of the CD experiment. The volume change was caused by evaporation of the solvent, despite efforts to prevent this, at high temperature. Therefore, we assume the concentration as well as the MRE values presented in the main text of the refolded sample are approximately 8% higher than reality. The first-order derivatives of the melting curves were calculated with Savitzky-Golay smoothing algorithm, and temperature where the minimum derivative value appears was regarded as the melting temperature. The molar residue ellipticity (MRE) value was calculated with the equation, MRE = (θ × m)/ (c × l × nr × 10) where θ represents the experimental ellipticity in millidegrees, m is the molecular weight of the peptide (g/mol), c is the peptide concentration (milligrams/milliliter), l is the path length of the cuvette (cm), and nr is the number of amino acid residues in the peptide.

CD spectrum

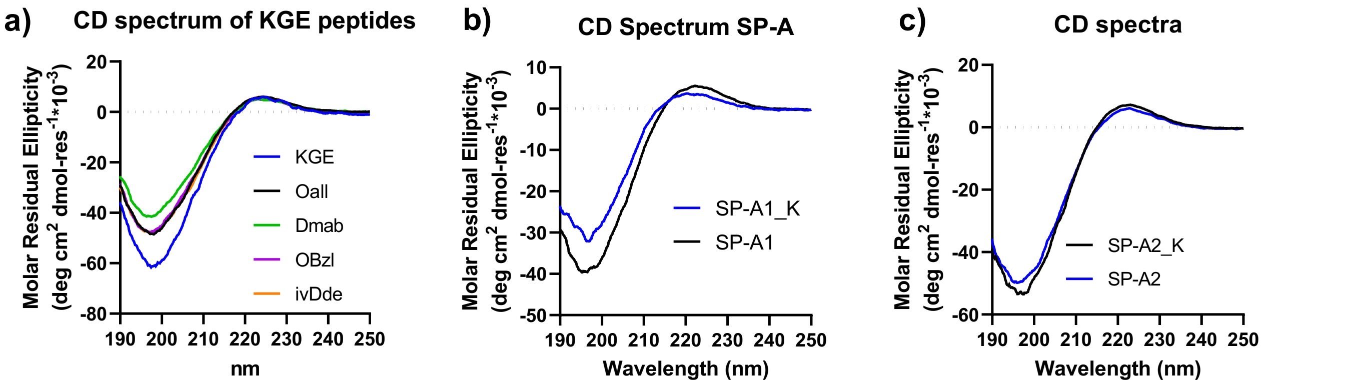

Figure S16. CD spectra of folded supramolecular triple helix. a) CD spectra of triple helices from unprotected and protected KGE peptides. b) CD spectra of triple helices from SP-A1 peptides with and without protecting groups. c) CD spectra of triple helices from unprotected and protected SP-A2 peptides.

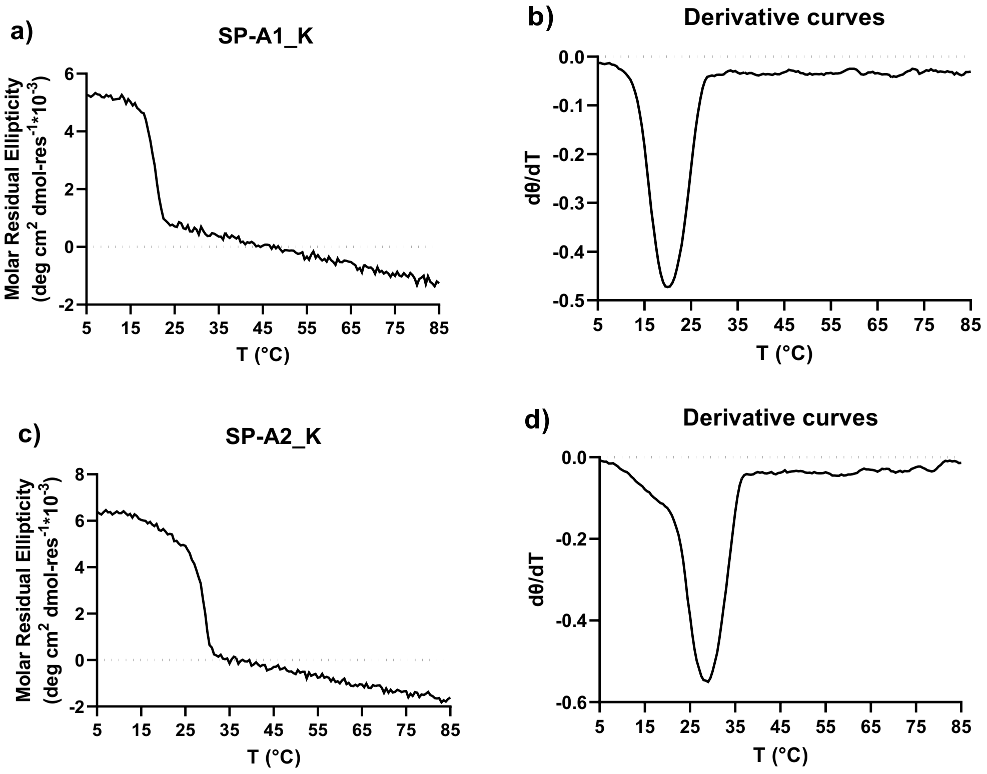

Figure S17. CD melting curves of supramolecular SP-A related triple helices with ivDde protecting groups. a) CD melt of supramolecular triple helix assembled from peptide SP-A1_K. b) first-order derivative of the melting curve in part a. c) CD melt of supramolecular triple helix assembled from peptide SP-A2_K. d) first-order derivative of the melting curve in part c.

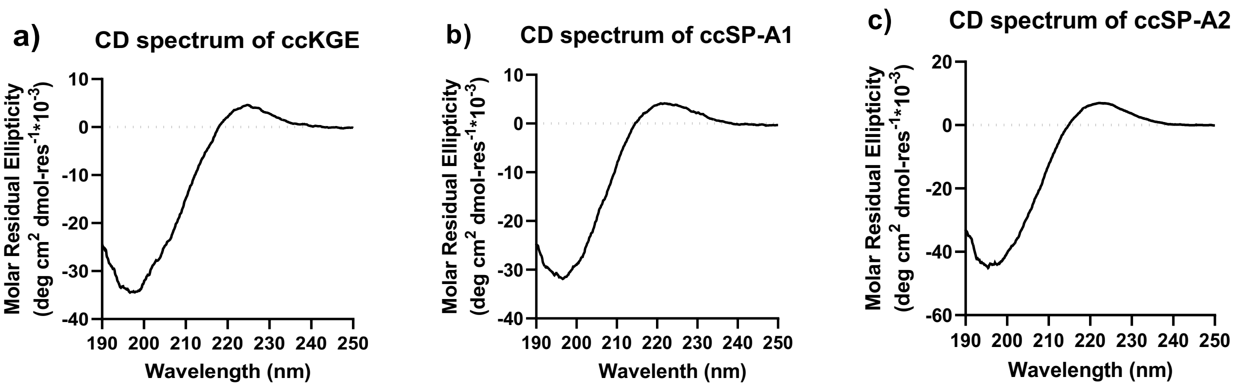

Figure S18. CD spectra of covalently captured triple helix. a) CD spectrum of covalently captured KGE trimer. b) CD spectrum of covalently captured SP-A1 trimer (three covalent bonds). c) CD spectrum of covalently captured SP-A2 trimer (six covalent bonds).

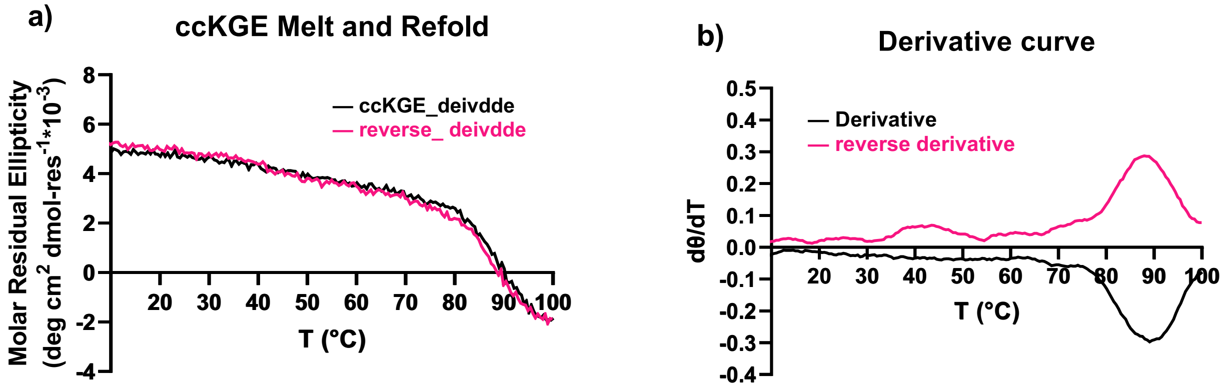

Figure S19. CD melt and refold curves of covalently captured triple helix. a) CD spectrum of covalently captured KGE trimer from ivDde protectibg group removal. b) Derivative curves of the corresponding melt and refold curves in part a.

References

(1) Hulgan, S. A. H.; Jalan, A. A.; Li, I.-C.; Walker, D. R.; Miller, M. D.; Kosgei, A. J.; Xu, W.; Phillips, G. N.; Hartgerink, J. D. Covalent Capture of Collagen Triple Helices Using Lysine–Aspartate and Lysine–Glutamate Pairs. *Biomacromolecules* **2020**, *21* (9), 3772–3781. https://doi.org/10.1021/acs.biomac.0c00878.

(2) Jbara, M.; Eid, E.; Brik, A. Palladium Mediated Deallylation in Fully Aqueous Conditions for Native Chemical Ligation at Aspartic and Glutamic Acid Sites. *Organic & Biomolecular Chemistry* **2018**, *16* (22), 4061–4064. https://doi.org/10.1039/C8OB00890F.
